# Cell Volume (3D) Correlative Microscopy Facilitated by Intracellular Fluorescent Nanodiamonds as Multi-Modal Probes

**DOI:** 10.3390/nano11010014

**Published:** 2020-12-23

**Authors:** Neeraj Prabhakar, Ilya Belevich, Markus Peurla, Xavier Heiligenstein, Huan-Cheng Chang, Cecilia Sahlgren, Eija Jokitalo, Jessica M. Rosenholm

**Affiliations:** 1 Pharmaceutical Sciences Laboratory, Faculty of Science and Engineering, Åbo Akademi University, 20520 Turku, Finland; jerosenh@abo.fi; 2 Cell Biology, Faculty of Science and Engineering, Åbo Akademi University, 20520 Turku, Finland; csahlgre@abo.fi; 3 Electron Microscopy Unit, Helsinki Institute of Life Science—Institute of Biotechnology, University of Helsinki, FI-00014 Helsinki, Finland; ilya.belevich@helsinki.fi (I.B.); eija.jokitalo@helsinki.fi (E.J.); 4 Institute of Biomedicine, Faculty of Medicine, University of Turku, 20520 Turku, Finland; markus.peurla@utu.fi; 5 Cancer Research Laboratory FICAN West, Institute of Biomedicine, University of Turku, 20520 Turku, Finland; 6 Turku Bioscience Centre, University of Turku and Åbo Akademi University, 20520 Turku, Finland; 7 CryoCapCell, 155 Boulevard de l’Hopital, 75013 Paris, France; xavier.heiligenstein@curie.fr; 8 Institute of Atomic and Molecular Sciences, Academia Sinica, Taipei 10617, Taiwan; hchang@sinica.edu.tw

**Keywords:** correlative microscopy, 3D CLEM, volume imaging

## Abstract

Three-dimensional correlative light and electron microscopy (3D CLEM) is attaining popularity as a potential technique to explore the functional aspects of a cell together with high-resolution ultrastructural details across the cell volume. To perform such a 3D CLEM experiment, there is an imperative requirement for multi-modal probes that are both fluorescent and electron-dense. These multi-modal probes will serve as landmarks in matching up the large full cell volume datasets acquired by different imaging modalities. Fluorescent nanodiamonds (FNDs) are a unique nanosized, fluorescent, and electron-dense material from the nanocarbon family. We hereby propose a novel and straightforward method for executing 3D CLEM using FNDs as multi-modal landmarks. We demonstrate that FND is biocompatible and is easily identified both in living cell fluorescence imaging and in serial block-face scanning electron microscopy (SB-EM). We illustrate the method by registering multi-modal datasets.

## 1. Introduction

Correlative light and electron microscopy (CLEM) combine the strengths of fluorescence and electron microscopy and this allows overcoming their respective limitations for cell imaging [1,2,3]. CLEM can be employed to study dynamics and localization of macromolecules and proteins with live cell light microscopy (LM) followed by electron microscopic (EM) examination of the ultrastructural morphology of the specific cell of interest [4,5,6,7,8,9]. Thus, functional and ultrastructural details of one cell are obtained by the integration of the two imaging modalities [10,11]. To date, numerous experimental CLEM approaches have been reported [5,12,13,14]. Apart from providing functional and ultrastructural information, recent CLEM methods have employed super-resolution fluorescence techniques to bridge the resolution gap between diffraction-limited fluorescence microscopy and EM [6,13,15,16,17,18]. However, the majority of developed CLEM methods are based on the correlation of LM with 2D images of thin cell sections imaged with transmission electron microscopy (TEM) [13,16,17,19,20,21]. Consequently, these CLEM methods provide very limited information on the *z*-axis direction, as TEM sections are generally restricted to slices of about 60–100 nm thickness and they may also be tilted relative to the image planes in the confocal image stack, resulting in uncertainty in the final correlation.

Considering the complex 3D organization of a cell, most of the critical 3D cellular information, especially in the z-direction, is generally under-explored. Therefore, 2D CLEM methods could be improved by employing instruments capable of performing 3D imaging [14,22,23,24,25,26,27,28,29], enabling CLEM methods to correlate 3D information from both LM and EM. Combining 3D fluorescence microscopy with 3D EM would significantly improve the technical possibilities for investigating complex cellular processes across the full volume of a cell [30].

Recently, several volume-CLEM methods that demonstrate 3D correlation have been presented [31,32,33,34,35,36]. Typically, there are common landmarks that are used as fiducials to facilitate correlation. These landmarks must be detectable with both imaging modalities. One such fluorescent and electron-dense CLEM marker is the fluorescent nanodiamond (FND) [19,37,38,39,40,41,42,43,44]. FNDs are non-toxic to cells, and being nanosized particles, they can be easily internalized in living cells via endocytosis [45,46,47,48]. FNDs have excellent photostability, and they have non-blinking far-red emission, which makes them well-suited for the imaging of living and fixed cells. We recently reported that FNDs are robust intracellular landmarks in 2D CLEM experiments [39].

In this article, a 3D CLEM method using on average 35 nm-sized FNDs as intracellular correlation landmarks for combining cell volume datasets from live-cell confocal microscopy and serial block-face scanning electron microscopy (SB-EM) is demonstrated.

## 2. Materials and Methods

### 2.1. FND Production

The synthesis and characterization of 35 nm FNDs have been previously reported [49]. A brief synthesis protocol is presented as follows. Synthetic type Ib diamond powders with a nominal size of 100 nm (MDA, Element Six) were purified in acids and suspended in water. A thin diamond film of ~50 μm thickness were made by depositing the diamond suspension on a silicon wafer. The diamond film was then treated by a 3-MeV proton beam and nitrogen-vacancy defect centers were created by annealing the proton beam-treated nanodiamonds. To produce 35 nm FNDs, the 100 nm FNDs were first mixed with NaF powders and crushed together with a hydraulic oil press under a pressure of 10 tons. Smaller FNDs were isolated by centrifugation after dissolving the mixture in hot water to remove NaF.

### 2.2. Cell Culture

MDA-MB-231 (Human breast adenocarcinoma) cells were obtained from Turku Bioscience Center, University of Turku and Åbo Akademi University, Finland. Cells were cultured in Dulbecco’s modified Eagle’s medium (DMEM (Lonza, Basel, Switzerland)) supplemented with 10% fetal bovine serum, 2 mM L-glutamine, and 1% penicillin-streptomycin (*v/v*), over µ-Dish 35 mm ibidi gridded dishes (ibidi GmbH, Gräfelfing, Germany). 10 µg/mL of 35 nm FNDs particles were prepared in 1 mL of cell growth media. Then, the cell media with particles was added to the cells growing. The cells were allowed to incubate with FNDs for 24 h. Staining with living cell dyes was performed as follows. The cells were washed three times with serum-free DMEM, after which 0.2 μL of Mitotracker (MitoTracker^®^ Green, ThermoFisher Scientific Inc., Waltham, MA, USA) was first added to 1.5 mL of medium (without serum and antibiotics) and then drop by drop to the dish. MDA-MB-231 cells were incubated for 30 min at 37 °C.

### 2.3. 2D SEM

MDA-MB-231 (Human breast adenocarcinoma) cells were cultured in Dulbecco’s modified Eagle’s medium (DMEM, Lonza, Basel, Switzerland) supplemented with 10% fetal bovine serum, 2 mM L-glutamine, and 1% penicillin-streptomycin (*v/v*). Of 35 nm FNDs particles, 10 µg/mL were prepared in 1 mL of cell growth media. Then, the cell media with particles was added to the cells growing. The cells were allowed to incubate with FNDs for 24 h. Cells were fixed with 5% glutaraldehyde s-collidine buffer, postfixed with 2% OsO_4_ containing 3% potassium ferrocyanide, dehydrated with ethanol, and flat embedded in a 45,359 Fluka Epoxy Embedding Medium kit. Thin sections were cut using an ultramicrotome to a thickness of 100 nm. The sections were stained using uranyl acetate and lead citrate to enable detection with SEM. The Zeiss LEO 1530 (Zeiss, Oberkochen, Germany) SEM instrument used was for imaging. The applied voltage was 15 kV, the detector was the in-lens detector. The secondary electron detector was placed in the electron optics column.

### 2.4. Confocal Microscopy

The living cell 3D imaging was performed with a Leica TCS SP5 confocal microscope (Leica Microsystems, Wetzlar, Germany), using a 63X oil objective. The cells were maintained at 37 °C, 5% CO_2_ during the imaging. The MitoTracker^®^ Green and the FNDs were excited by 488 nm argon laser. Fluorescence was collected at 510–550 nm and 650–730 nm with PMTs (Photomultiplier tubes) for MitoTracker^®^ Green and FNDs, respectively. The MitoTracker^®^ Green was recorded in 3D stacks together with FND landmarks in living cells. Live cell microscopy was performed for 2.5 min to obtain 35 stacks of step size 0.13 µm. The live cells were instantly fixed in a fixative mixture consisting of 2% glutaraldehyde, 2% PFA, 2 mM CaCl_2_ in 0.1 M NaCac buffer, pH 7.4.

### 2.5. 3D SB-EM Sample Preparation

The specimens were prepared using a protocol modified from Deerinck et al. (2010) [50]. The cells were fixed for 30 min at RT with a fixative mixture consisting of 2% glutaraldehyde, 2% PFA, 2 mM CaCl_2_ in 0.1 M NaCac buffer (pH 7.4) and washed five times with NaCac buffer containing 2 mM CaCl_2_. The cells were postfixed for 1 h on an ice bath in a fume hood with 2% OsO, 1.5% K_4_[Fe(CN)_6_], 2 mM CaCl_2_ in 0.1 M NaCac buffer, pH 7.4. The cells were washed 5 times with distilled water (DW). The cells were then incubated in 1% aqueous thiocarbohydrazide (TCH) for 10 min at RT. The cells were washed 5 times with DW. The cells were incubated in 1% OsO_4_ in DW for 30 min at RT. The cells were washed 5 times with DW. The cells were incubated with 1% uranyl acetate at +4 °C overnight, washed 5 times with DW at RT, incubated in the pre-warmed lead aspartate solution at 60˚C oven for 30 min., and washed 5 times with DW followed by serial dehydration. The cells were dipped into an aluminium plate with a resin-acetone solution containing acetone with 50% (*v/v*) Epon resin to incubate for 1 h. Further, cells were incubated in 100% Epon resin and incubated for 1 h RT. The cells were allowed to polymerize in an oven at 60 °C for 28 h.

### 2.6. 3D SB-EM Imaging

The area of interest with the selected cells was trimmed from the plastic block and mounted onto a pin using conductive epoxy glue (model 2400; CircuitWorks, Kennesaw, GA, USA). The trimmed block was further trimmed as a pyramid and its sides were covered with silver paint (Agar Scientific Ltd., Stansted, UK). To improve conductivity, the whole assembly was platinum-coated using Quorum Q150TS (Quorum Technologies, Laughton, UK). SB-EM data sets were acquired with a FEG-SEM Quanta 250 (Thermo Fisher Scientific, FEI, Hillsboro, OR, USA), using a backscattered electron detector (Gatan Inc., Pleasanton, CA, USA) with 2.5-kV beam voltage, a spot size of 2.9, and a pressure of 0.15 Torr. The block faces were cut with 50-nm increments and imaged with XY resolution of 25 nm per pixel. The collected 16-bit images were processed for segmentation using an open-source software Microscopy Image Browser [51] as follows: (a) individual images were combined into 3D stacks; (b) the combined 3D-stack was aligned; (c) the contrast for the whole stack was adjusted, and (d) the images were converted to the 8-bit format.

### 2.7. Image Correlation

The multi-modal datasets were registered using the eC-CLEM plugin on the Icy bioimage analysis platform [52]. To match the large datasets on a laptop (i7, 16Gb RAM), the EM stack was binned 4 times. The FM stack was matched to the binned dataset using the FNDs as landmarks, targeting the center of the FNDs aggregates both in LM and EM using orthogonal views from Icy. Nine FNDs were sufficient to achieve the good overlay accuracy depicted in this manuscript. Rigid registration was performed despite a recommendation by the software to apply for non-rigid registration [53]. This decision was made after careful observation of the LM dataset. Since living cell imaging was performed on the 3D stack, the cell dynamics caused some of the FNDs to move during image acquisition. This natural movement was uneven in all FNDs. Local inaccuracies in this registration were coherent with the cell movement observed. The weighing of each landmark operated by eC-CLEM compensated for the shifts observed between the LM and the EM dataset and rigid registration lead to accurate full registration. To generate the final overlay, the transformation was applied to the LM dataset to match the original EM dataset using the “apply a reduced scaled transform to a full-size image” function from eC-CLEM (Advanced usage). This final overlay was used to generate movies in supplementary data.

## 3. Results

### FND Facilitated 3D Cell Volume-CLEM

**Figure 1:**
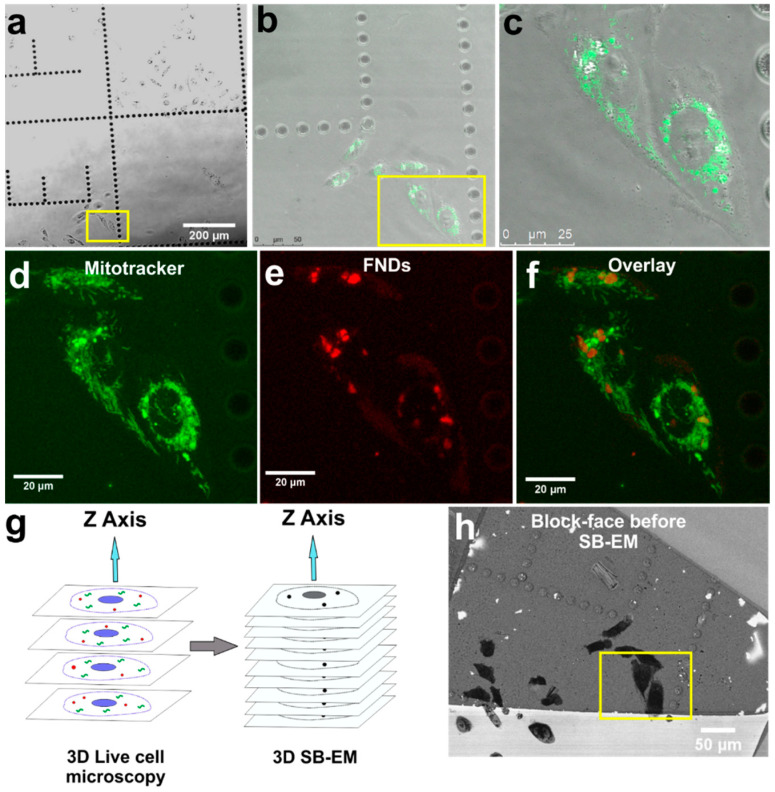
The 3D correlative light and electron microscopy workflow demonstrating the live-cell confocal microscopy to the serial block-face scanning electron microscopy (SB-EM). (**a**) Bright-field image of the living cells on a gridded glass-bottom dish. The cells selected for 3D CLEM are indicated by the yellow box. (**b**) MitoTracker signal from the stained selected cells (indicated by the yellow box). (**c**) Close-up view of the selected cells shown by an overlay of brightfield and MitoTracker images. (**d**) Maximum intensity z-projection image from the MitoTracker channel of the selected cells. (**e**) Maximum intensity z-projection image from the fluorescent nanodiamond (FND) channel of the selected cells. (**f**) Overlay of maximum intensity z-projections of MitoTracker and FND channels. (**g**) Schematic representation of 3D CLEM workflow with FNDs (red dots) and a standard organic fluorophore (green structures). (**h**) Localization of the selected cells on the EM block-face (near the letter E) for SB-EM. The yellow box indicates the same selected cells as in (**a**).

Our 3D CLEM workflow begins by seeding FND incubated MDA-MB-231 cells over gridded glass-bottom dishes designed for CLEM experiments. Two FND incubated living MDA-MB-231 cells shown in Figure 1a were selected for the 3D CLEM experiment. To demonstrate the usefulness of FNDs in the 3D CLEM experiment, MDA-MB-231 cells were stained with a mitochondrial marker dye MitoTracker (Figure 1b,c).

Confocal image stacks of the whole cell volumes were acquired from both the MitoTracker (green) and the FND (red) signals (Figure 1d–f and Video S1). MitoTracker signal was seen widespread in cytoplasmic space (Figure 1d). The fluorescence signal from FNDs (Figure 1e) was mostly localized to a few spots suggesting their confinement in vesicles. Following earlier results FNDs are internalized by clathrin-mediated endocytosis [45,46] and they tend to aggregate inside endosomal vesicles (Figure S1) and subsequently slowly exocytose from cells [45,47]. The aggregation of FNDs in cellular vesicles brings added benefit from a CLEM perspective [39] because, in comparison to single FNDs, the high concentration of FNDs aggregated inside vesicles provides better contrast both in fluorescence microscopy and in EM. Besides, the confinement of FNDs in vesicles prevents their movement in the sample processing steps after the confocal imaging enabling more reliable correlation of EM images.

3D localization of FNDs for the MitoTracker fluorescence signal can be seen in Video S2. These 3D confocal datasets were used for software-based correlation with SB-EM datasets. After confocal imaging, the selected cells were fixed, stained, and embedded for SB-EM (Figure 1g). The use of gridded glass-bottom dishes allowed easy identification of the cells of interest within the plastic block and trimming the blocks accordingly. The trimmed area was mounted on a pin and imaged in SEM. The mounted block-face overview image before SB-EM is displayed in Figure 1h.

**Figure 2:**
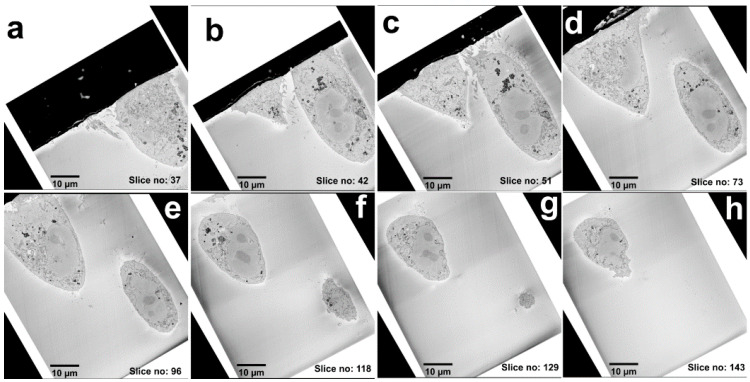
A collage of multi-plane images from the SB-EM 3D series. (**a**–**h**) Images from the 3D EM stack acquired from the selected cells are shown in the increasing order of the *z*-axis. The distance between the slices is 50 nm.

The two selected cells were identified (Figure 1h) using a 15 kV electron beam. The collection of 3D EM data was performed with an SEM instrument equipped with a system for serial block-face SEM (SB-EM). In SB-EM, an ultramicrotome performed automated sectioning of whole-cell volume by cutting thin sections (≥50 nm) from the sample’s block-face (Video S3). Consequently, after each cut, a high-resolution image of the freshly made block-face was acquired using a backscattered electron detector to form a 3D image stack. SB-EM imaging provided a three-dimensional dataset of the selected cells with a resolution to recognize the structure of interest (mitochondria) and FNDs aggregated in vesicular structures for CLEM (Figure 2).

**Figure 3:**
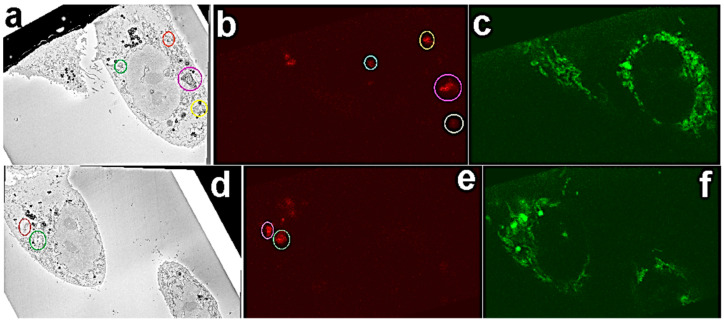
Overview of the corresponding FND aggregates in EM and LM. Corresponding FNDs (color-coded) were matched in respective EM and light microscopy (LM) sections. (**a**,**d**) SEM images with FND localized in vesicles (color-coded). (**b**,**e**) Respective FNDs (color-coded) in LM images were matched up. (**c**,**f**) corresponding MitoTracker channels.

Correlation of the LM and SB-EM volume datasets was done using the eC-CLEM plugin on the Icy bioimage analysis platform [52,54]. First corresponding intracellular FNDs were identified in both datasets. FNDs aggregated in vesicles have a distinct appearance in SEM images (Supplementary Figure S1) and they are easily distinguished from morphological features of the cell. Figure 3 shows representative SB-EM and 3D LM image pairs (Figure 3a,b; Figure 3d,e) in which the corresponding FNDs are identified and marked.

**Figure 4:**
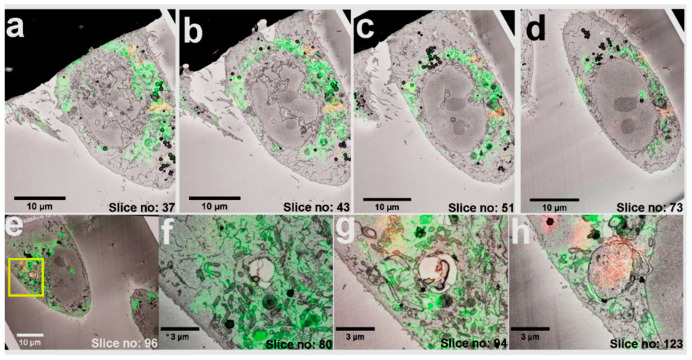
Volume-CLEM of living cells. (**a**–**d**) CLEM images of multiple planes in increasing *z*-axis. (**e**) ROI selected in CLEM image to demonstrate the correlation of LM over EM. (**f**) In slice no: 80, a single high-resolution image shows numerous mitochondria (green) and an empty vesicle. (**g**) In slice no: 94, FNDs (red), mitochondria (green), and a vesicle can be seen. (**h**) In slice no: 123, a vesicle filled with FNDs (red), and fewer mitochondria (green) can be seen. FNDs in the EM image are the dark dots inside the vesicle.

Correlation of the two volume datasets was calculated using the identified FND position pairs as fiducials, and the accordingly transformed volume dataset of the fluorescence signal of interest (MitoTracker) was overlayed on the SB-EM stack. The FNDs facilitated mapping of the mitochondrial locations throughout the cell volume (Figure 4a–d) resulting in good colocalization of the MitoTracker signal with mitochondria seen in the EM image stack (Videos S4–S6 and Figure S2).

Quite commonly in the literature, the multi-modal correlation is performed in absence of such a common landmark, and the process of 3D dataset correlation severely suffers from misalignment and errors in localizing critical information across 3D. However, in this type of CLEM approach, there can be multiple factors that could affect precise image correlation. The major challenge encountered in the CLEM experiment was inherently low axial (600 nm) and lateral (250 nm) resolution provided by confocal microscopes compared to the nanometer scale resolution provided by EM (Supplementary Figure S3). Currently, the limited resolution of confocal microscopy can result in the misalignment of details within large scale datasets. Sample autofluorescence, unspecific binding of fluorophores, and obtaining a bright FND signal with live-cell imaging are additional parameters that still must be optimized.

## 4. Discussion

We have introduced a novel FND enabled cell volume (3D) correlative microscopy method. The CLEM workflow is straightforward and can be performed without any dedicated CLEM imaging systems. We demonstrated that a standard organic fluorophore can be used for 3D CLEM experiments with the FND-based method without any special sample preparation requirements.

In general, organic fluorophores do not survive routine EM sample processing and are not electron dense molecules, and therefore are not detectable with EM. In contrast, the employed 35 nm FNDs were intracellularly detectable with both imaging modalities in our experiments, enabling the successful correlation of volume datasets for 3D CLEM. FNDs can offer multiple advantages over currently used CLEM fiducials as their internalization does not need chemical permeabilization, which has impacts on cellular morphology and ultrastructure. FNDs may be considered as a leading contender in the search for an exceptional CLEM probe because they are not prone to chemical degradation, have excellent photostability, and their nanoscale size facilitates their rapid internalization to cells.

In our CLEM workflow, confocal microscopy was chosen for 3D living cell imaging even if it offers a limited resolution. Pairing confocal with SB-EM imaging was a practical choice for our experiment because the specific instrument was available to us. However, the focused ion beam imaging (FIB-SEM) could be used as an alternative for automatically obtaining the serial section image stacks. However, SB-EM can manage larger sample volumes than FIB-SEM, but with more limited z resolution. Our next step is to explore the possibilities of performing FND-enabled CLEM with 3D super-resolution imaging.

## Data Availability

The data presented in this study are openly available in [Zenodo] at [doi.org/10.5281/zenodo.4384502].

## Supplementary Materials

The following are available online at https://www.mdpi.com/2079-4991/11/1/14/s1. Figure S1: SEM imaging of vesicle aggregated FNDs; Figure S2: FND landmarks facilitated the alignment of 3D images; Figure S3: Single stacks image correlation; Video S1: 3D live cell confocal microscopy; Video S2: 3D reconstruction of FND distribution; Video S3: 3D SB-EM imaging of both cells; Video S4: 3D CLEM of both cells; Video S5: 3D CLEM of vesicle aggregated FND; Video S6: 3D CLEM of single cell. The video files referred to in the article as Videos S1–S6 are available on Zenodo at https://zenodo.org/record/4279702 (DOI:10.5281/zenodo.4279702).

## References

[1] Mironov A.A., Beznoussenko G.V. (2012). Correlative light-electron microscopy: A potent tool for the imaging of rare or unique cellular and tissue events and structures. Methods Enzymol..

[2] de Boer P., Hoogenboom J.P., Giepmans B.N.G. (2015). Correlated light and electron microscopy: Ultrastructure lights up!. Nat. Methods.

[3] Sartori A., Gatz R., Beck F., Rigort A., Baumeister W., Plitzko J.M. (2007). Correlative microscopy: Bridging the gap between fluorescence light microscopy and cryo-electron tomography. J. Struct. Biol..

[4] Polishchuk E.V., Polishchuk R.S., Luini A., Taatjes D., Roth J. (2012). Correlative Light–Electron Microscopy as a Tool to Study in Vivo Dynamics and Ultrastructure of Intracellular Structures. Methods in Molecular Biology (Methods and Protocols).

[5] Polishchuk E.V., Polishchuk R.S. (2018). Pre-embedding labeling for subcellular detection of molecules with electron microscopy. Tissue Cell.

[6] Watanabe S., Punge A., Hollopeter G., Willig K.I., Hobson R.J., Davis M.W., Hell S.W., Jorgensen E.M. (2011). Protein localization in electron micrographs using fluorescence nanoscopy. Nat. Methods.

[7] Kopek B.G., Shtengel G., Xu C.S., Clayton D.A., Hess H.F. (2012). Correlative 3D superresolution fluorescence and electron microscopy reveal the relationship of mitochondrial nucleoids to membranes. Proc. Natl. Acad. Sci. USA.

[8] Razi M., Tooze S.A. (2009). Chapter 17 Correlative Light and Electron Microscopy. Methods Enzymol..

[9] Loussert Fonta C., Humbel B.M. (2015). Correlative microscopy. Arch. Biochem. Biophys..

[10] Smith C. (2012). Microscopy: Two microscopes are better than one. Nature.

[11] van Rijnsoever C., Oorschot V., Klumperman J. (2008). Correlative light-electron microscopy (CLEM) combining live-cell imaging and immunolabeling of ultrathin cryosections. Nat. Methods.

[12] Ando T., Bhamidimarri S.P., Brending N., Colin-York H., Collinson L., De Jonge N., De Pablo P.J., Debroye E., Eggeling C., Franck C. (2018). The 2018 correlative microscopy techniques roadmap. J. Phys. D Appl. Phys..

[13] Johnson E., Seiradake E., Jones E.Y., Davis I., Grünewald K., Kaufmann R. (2015). Correlative in-resin super-resolution and electron microscopy using standard fluorescent proteins. Sci. Rep..

[14] Peddie C.J., Collinson L.M. (2014). Exploring the third dimension: Volume electron microscopy comes of age. Micron.

[15] Joosten B., Willemse M., Fransen J., Cambi A., van den Dries K. (2018). Super-Resolution Correlative Light and Electron Microscopy (SR-CLEM) Reveals Novel Ultrastructural Insights into Dendritic Cell Podosomes. Front. Immunol..

[16] Peddie C.J., Domart M.-C., Snetkov X., O’Toole P., Larijani B., Way M., Cox S., Collinson L.M. (2017). Correlative super-resolution fluorescence and electron microscopy using conventional fluorescent proteins in vacuo. J. Struct. Biol..

[17] Wolff G., Hagen C., Grünewald K., Kaufmann R. (2016). Towards correlative super-resolution fluorescence and electron cryo-microscopy. Biol. Cell.

[18] Johnson E., Kaufmann R. (2017). Preserving the photoswitching ability of standard fluorescent proteins for correlative in-resin super-resolution and electron microscopy. Methods Cell Biol..

[19] Hemelaar S.R., de Boer P., Chipaux M., Zuidema W., Hamoh T., Martinez F.P., Nagl A., Hoogenboom J.P., Giepmans B.N.G., Schirhagl R. (2017). Nanodiamonds as multi-purpose labels for microscopy. Sci. Rep..

[20] Paez-Segala M.G., Sun M.G., Shtengel G., Viswanathan S., Baird M.A., Macklin J.J., Patel R., Allen J.R., Howe E.S., Piszczek G. (2015). Fixation-resistant photoactivatable fluorescent proteins for CLEM. Nat. Methods.

[21] Liv N., Zonnevylle A.C., Narvaez A.C., Effting A.P.J., Voorneveld P.W., Lucas M.S., Hardwick J.C., Wepf R.A., Kruit P., Hoogenboom J.P. (2013). Simultaneous Correlative Scanning Electron and High-NA Fluorescence Microscopy. PLoS ONE.

[22] Biazik J., Vihinen H., Anwar T., Jokitalo E., Eskelinen E.-L. (2015). The versatile electron microscope: An ultrastructural overview of autophagy. Methods.

[23] Nathans J., Hopkins J., Shan Xu C., Hayworth K.J., Lu Z., Grob P., Hassan A.M., García-Cerdá J.G., Niyogi K.K., Nogales E. (2017). Enhanced FIB-SEM systems for large-volume 3D imaging. eLife.

[24] Denk W., Horstmann H. (2004). Serial Block-Face Scanning Electron Microscopy to Reconstruct Three-Dimensional Tissue Nanostructure. PLoS Biol..

[25] Knott G., Marchman H., Wall D., Lich B. (2008). Serial section scanning electron microscopy of adult brain tissue using focused ion beam milling. J. Neurosci..

[26] Webb R., Webb R. (2015). Quick Freeze Substitution Processing of Biological Samples for Serial Block-face Scanning Electron Microscopy. Microsc. Microanal..

[27] Ghosh S., Tran K., Delbridge L.M.D., Hickey A.J.R., Hanssen E., Crampin E.J., Rajagopal V. (2018). Insights on the impact of mitochondrial organisation on bioenergetics in high-resolution computational models of cardiac cell architecture. PLoS Comput. Biol..

[28] Hussain A., Ghosh S., Kalkhoran S.B., Hausenloy D.J., Hanssen E., Rajagopal V. (2018). An automated workflow for segmenting single adult cardiac cells from large-volume serial block-face scanning electron microscopy data. J. Struct. Biol..

[29] Kremer A., Lippens S., Bartunkova S., Asselbergh B., Blanpain C., Fendrych M., Goossens A., Holt M., Janssens S., Krols M. (2015). Developing 3D SEM in a broad biological context. J. Microsc..

[30] Deerinck T.J., Shone T.M., Bushong E.A., Ramachandra R., Peltier S.T., Ellisman M.H. (2018). High-performance serial block-face SEM of nonconductive biological samples enabled by focal gas injection-based charge compensation. J. Microsc..

[31] Russell M.R.G., Lerner T.R., Burden J.J., Nkwe D.O., Pelchen-Matthews A., Domart M.-C., Durgan J., Weston A., Jones M.L., Peddie C.J. (2017). 3D correlative light and electron microscopy of cultured cells using serial blockface scanning electron microscopy. J Cell Sci.

[32] Bosch C., Martínez A., Masachs N., Teixeira C.M., Fernaud I., Ulloa F., Pérez-Martínez E., Lois C., Comella J.X., DeFelipe J. (2015). FIB/SEM technology and high-throughput 3D reconstruction of dendritic spines and synapses in GFP-labeled adult-generated neurons. Front. Neuroanat..

[33] Beckwith M.S., Beckwith K.S., Sikorski P., Skogaker N.T., Flo T.H., Halaas Ø. (2015). Seeing a Mycobacterium-Infected Cell in Nanoscale 3D: Correlative Imaging by Light Microscopy and FIB/SEM Tomography. PLoS ONE.

[34] Booth D.G., Beckett A.J., Molina O., Samejima I., Masumoto H., Kouprina N., Larionov V., Prior I.A., Earnshaw W.C. (2016). 3D-CLEM Reveals that a Major Portion of Mitotic Chromosomes Is Not Chromatin. Mol. Cell.

[35] Lucas M.S., Günthert M., Gasser P., Lucas F., Wepf R. (2012). Bridging Microscopes: 3D Correlative Light and Scanning Electron Microscopy of Complex Biological Structures. Methods in Cell Biology.

[36] Lucas M.S., Guenthert M., Gasser P., Lucas F., Wepf R. (2014). Correlative 3D imaging: CLSM and FIB-SEM tomography using high-pressure frozen, freeze-substituted biological samples. Methods in Molecular Biology.

[37] Hsieh F.-J., Chen Y.-W., Huang Y.-K., Lee H.-M., Lin C.-H., Chang H.-C. (2018). Correlative Light-Electron Microscopy of Lipid-Encapsulated Fluorescent Nanodiamonds for Nanometric Localization of Cell Surface Antigens. Anal. Chem..

[38] Han S., Raabe M., Hodgson L., Mantell J., Verkade P., Lasser T., Landfester K., Weil T., Lieberwirth I. (2019). High-Contrast Imaging of Nanodiamonds in Cells by Energy Filtered and Correlative Light-Electron Microscopy: Toward a Quantitative Nanoparticle-Cell Analysis. Nano Lett..

[39] Prabhakar N., Peurla M., Koho S., Deguchi T., Näreoja T., Chang H.-C., Rosenholm J.M., Hänninen P.E. (2018). STED-TEM Correlative Microscopy Leveraging Nanodiamonds as Intracellular Dual-Contrast Markers. Small.

[40] Schrand A.M., Hens S.A.C., Shenderova O.A. (2009). Nanodiamond Particles: Properties and Perspectives for Bioapplications. Crit. Rev. Solid State Mater. Sci..

[41] Vlasov I.I., Shiryaev A.A., Rendler T., Steinert S., Lee S.-Y., Antonov D., Vörös M., Jelezko F., Fisenko A.V., Semjonova L.F. (2014). Molecular-sized fluorescent nanodiamonds. Nat. Nanotechnol..

[42] Mochalin V.N., Shenderova O., Ho D., Gogotsi Y. (2011). The properties and applications of nanodiamonds. Nat. Nanotechnol..

[43] Prabhakar N., Näreoja T., Von Haartman E., Karaman D.Ş., Jiang H., Koho S., Dolenko T.A., Hänninen P.E., Vlasov D.I., Ralchenko V.G. (2013). Core-shell designs of photoluminescent nanodiamonds with porous silica coatings for bioimaging and drug delivery II: Application. Nanoscale.

[44] Hui Y.Y., Cheng C.-L., Chang H.-C. (2010). Nanodiamonds for optical bioimaging. J. Phys. D Appl. Phys..

[45] Chang H.-C., Li C.-L., Cheng C.-A., Chang C.-F., Yeh S.-H., Fang C.-Y., Vaijayanthimala V. (2011). The Exocytosis of Fluorescent Nanodiamond and Its Use as a Long-Term Cell Tracker. Small.

[46] Vaijayanthimala V., Tzeng Y.-K., Chang H.-C., Li C.-L. (2009). The biocompatibility of fluorescent nanodiamonds and their mechanism of cellular uptake. Nanotechnology.

[47] Prabhakar N., Khan M.H., Peurla M., Chang H.-C., Hänninen P.E., Rosenholm J.M. (2017). Intracellular Trafficking of Fluorescent Nanodiamonds and Regulation of Their Cellular Toxicity. ACS Omega.

[48] Prabhakar N., Rosenholm J.M. (2019). Nanodiamonds for advanced optical bioimaging and beyond. Curr. Opin. Colloid Interface Sci..

[49] Su L.-J., Fang C.-Y., Chang Y.-T., Chen K.-M., Yu Y.-C., Hsu J.-H., Chang H.-C. (2013). Creation of high density ensembles of nitrogen-vacancy centers in nitrogen-rich type Ib nanodiamonds. Nanotechnology.

[50] Deerinck T.J., Bushong E., THOR A., Ellisman M., Deerinck T., Thor A., Deerinck T., Bushong E., Thor C.A., Ellisman M. (2010). NCMIR methods for 3D EM: A new protocol for preparation of biological specimens for serial block face scanning electron microscopy. Microscopy.

[51] Belevich I., Joensuu M., Kumar D., Vihinen H., Jokitalo E. (2016). Microscopy Image Browser: A Platform for Segmentation and Analysis of Multidimensional Datasets. PLoS Biol..

[52] Paul-Gilloteaux P., Heiligenstein X., Belle M., Domart M.-C., Larijani B., Collinson L., Raposo G., Salamero J. (2017). eC-CLEM: Flexible multidimensional registration software for correlative microscopies. Nat. Methods.

[53] Prabhakar N., Peurla M., Shenderova O., Rosenholm J.M. (2020). Fluorescent and Electron-Dense Green Color Emitting Nanodiamonds for Single-Cell Correlative Microscopy. Molecules.

[54] de Chaumont F., Dallongeville S., Chenouard N., Hervé N., Pop S., Provoost T., Meas-Yedid V., Pankajakshan P., Lecomte T., Le Montagner Y. (2012). Icy: An open bioimage informatics platform for extended reproducible research. Nat. Methods.

