# Supplementary material for: Cell Volume (3D) Correlative Microscopy Facilitated by Intracellular Fluorescent Nanodiamonds as Multi-Modal Probes
Source: Nanomaterials (Basel). 2020 Dec 23;11(1):14. doi: 10.3390/nano11010014 (PMC7822478; doi:10.3390/nano11010014)
Supplement: Supplementary file 1 [file nanomaterials-11-00014-s001.pdf]

nanomaterials

MDPI

Supplementary Materials

Cell Volume (3D) Correlative Microscopy Facilitated by Intracellular Fluorescent Nanodiamonds as Multi-Modal Probes

Neeraj Prabhakar [1,2,*], Ilya Belevich [3], Markus Peurla [4,5,6], Xavier Heiligenstein [7], Huan-Cheng Chang [8], Cecilia Sahlgren [2], Eija Jokitalo [3] and Jessica M. Rosenholm [1]

1 Pharmaceutical Sciences Laboratory, Faculty of Science and Engineering, Åbo Akademi University, 20520 Turku, Finland; jerosenh@abo.fi
2 Cell Biology, Faculty of Science and Engineering, Åbo Akademi University, 20520 Turku, Finland; csahlgre@abo.fi
3 Electron Microscopy Unit, Helsinki Institute of Life Science—Institute of Biotechnology, University of Helsinki, FI-00014 Helsinki, Finland; ilya.belevich@helsinki.fi (I.B.); eija.jokitalo@helsinki.fi (E.J.)
4 Institute of Biomedicine, Faculty of Medicine, University of Turku, 20520 Turku, Finland; markus.peurla@utu.fi
5 Cancer Research Laboratory FICAN West, Institute of Biomedicine, University of Turku, 20520 Turku, Finland
6 Turku Bioscience Centre, University of Turku and Åbo Akademi University, 20520 Turku, Finland
7 CryoCapCell, 155 Boulevard de l'Hopital, 75013 Paris, France; xavier.heiligenstein@curie.fr
8 Institute of Atomic and Molecular Sciences, Academia Sinica, Taipei 10617, Taiwan; hchang@sinica.edu.tw
* Correspondence: nprabhak@abo.fi

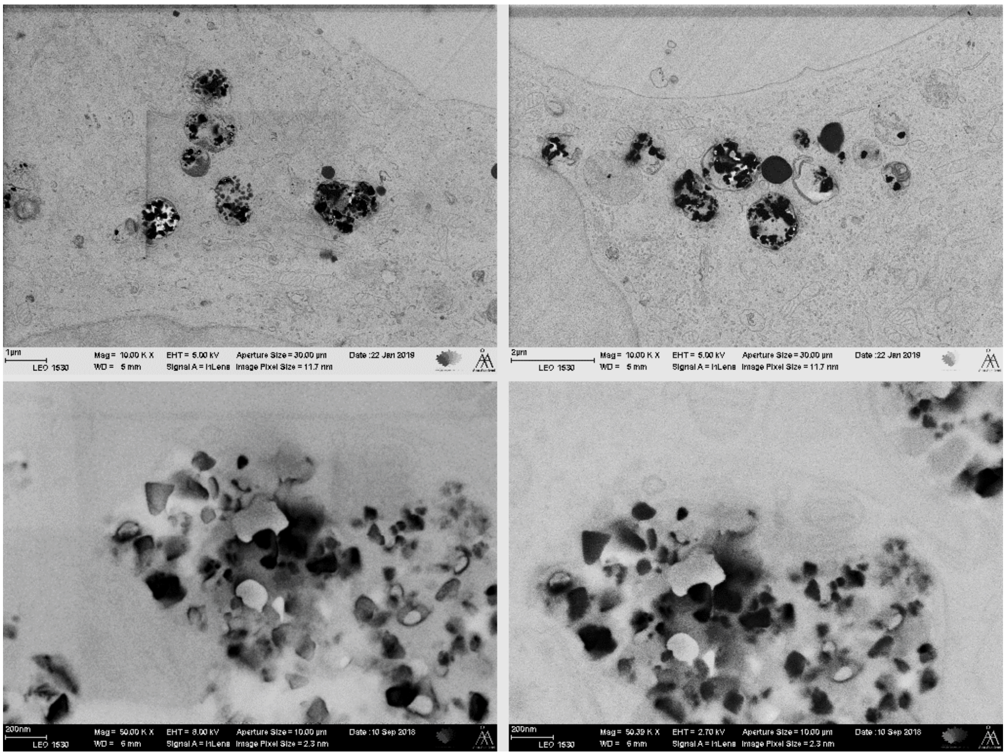

Figure S1. A thin 100 nm cell section imaged with SEM. The Zeiss LEO 1530 (Zeiss, Germany) SEM instrument used was for imaging. FNDs were aggregated in vesicles of different sizes ranging from 0.5–2µm. Vesicular localization of FNDs provides high contrast for imaging.

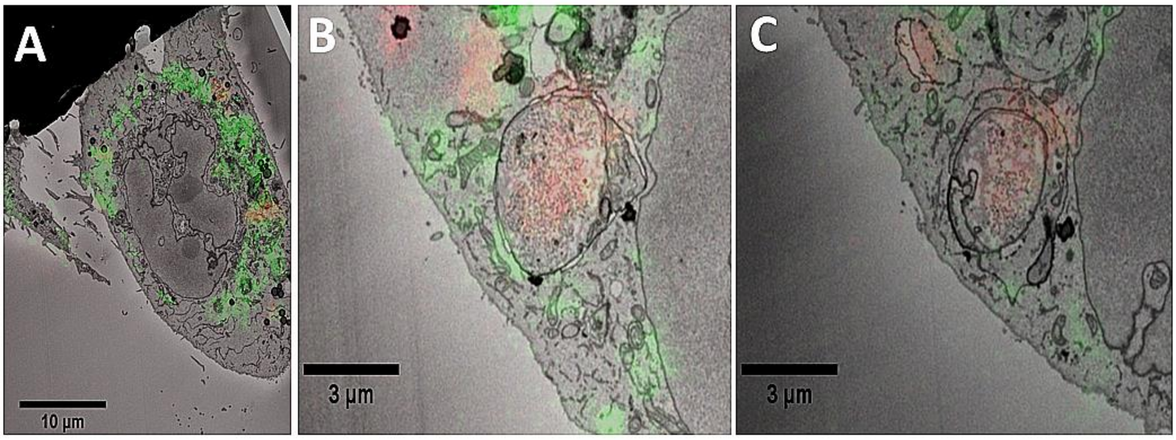

Figure S2. FNDs landmarks facilitated the alignment of the MitoTracker channel over SB-EM stacks. We have demonstrated here the image alignment across different Z planes. **A)** FNDs (red) and Mitotracker (green) are shown overlaid over SB-EM images and vesicle aggregated FNDs can be seen as well**. B)** A large FNDs vesicle correlated with complementary fluorescence data from same planes. **C)** Corresponding SEM and LM sections alignment.

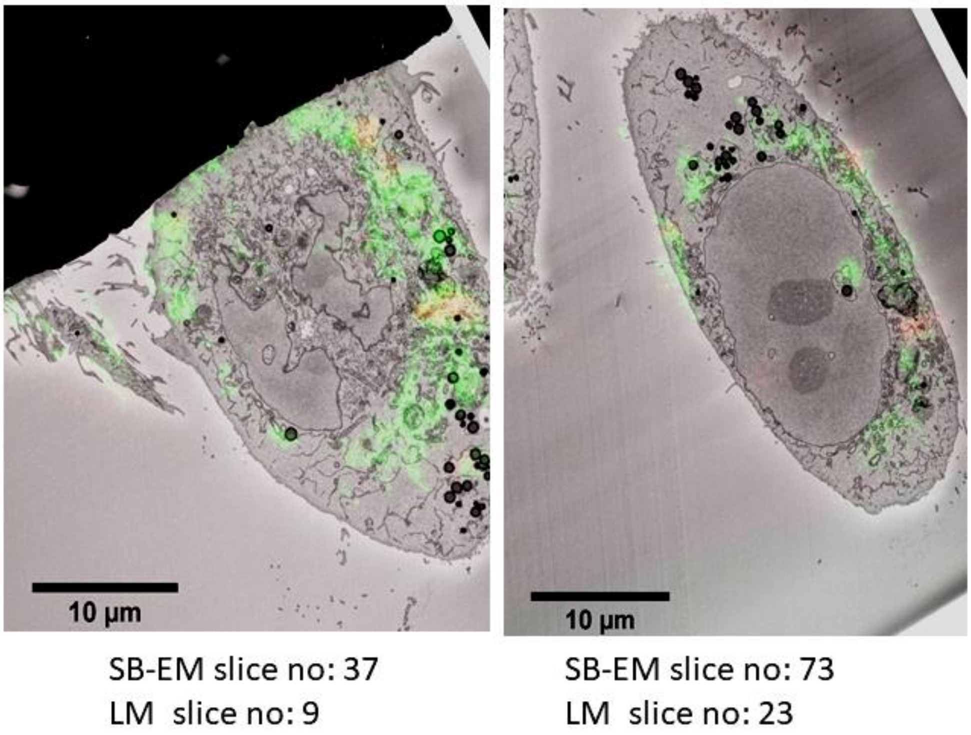

Figure S3. The major challenge encountered in the CLEM experiment was inherently low axial (600 nm) and lateral (250 nm) resolution provided by confocal microscopes compared to the nanometer scale resolution provided by EM. Currently, the limited resolution of confocal microscopy can result in the misalignment of details within large scale datasets, especially across the Z-axis.

Supplementary Video Files 1–6. The video files referred to in the article as **Video 1–6** are available on Zenodo at https://zenodo.org/record/4279702 (DOI: 10.5281/zenodo.4279702)

© 2020 by the authors. Licensee MDPI, Basel, Switzerland. This article is an open access article distributed under the terms and conditions of the Creative Commons Attribution (CC BY) license (http://creativecommons.org/licenses/by/4.0/).
